# Unrecognized Orbital Images Cause Diagnostic Confusion: Silicone Oil and Implanted Silicone Encircling Bands

**DOI:** 10.1155/2021/9940395

**Published:** 2021-06-21

**Authors:** Tsuyoshi Nojima, Takafumi Obara, Kohei Tsukahara, Atsunori Nakao, Hiromichi Naito

**Affiliations:** Department of Emergency, Critical Care and Disaster Medicine, Okayama University Graduate School of Medicine, Dentistry and Pharmaceutical Sciences, Okayama, Japan

## Abstract

**Introduction:**

Most physicians are not familiar with postoperative changes to the orbit, so radiologists and clinicians may sometimes find it challenging to conduct a proper radiological assessment of the globe of the eye and orbital abnormalities. We present a patient with head trauma who had surgery for retinal detachment with implantation of silicone encircling bands. This case report may help clinicians recognize imaging characteristics after ophthalmic surgery to prevent misdiagnosis and unnecessary workup. *Case Report*. An 18-year-old man with severe head trauma was admitted to our hospital. Initial computed tomography (CT) revealed a high attenuation of intraocular silicone that could be mistaken for a hemorrhage. Ophthalmological examination and detailed ophthalmic history confirmed silicone oil in his eye for treatment of retinal detachment. Knowledge of the anatomical changes and radiological appearance of postsurgical findings following retinal detachment, including the surgical materials of silicone oil or bands, can prevent unnecessary alarm.

**Conclusion:**

Implanted ophthalmic devices, for example, silicone oil, appear similar to hemorrhages on CT and magnetic resonance imaging and cause diagnostic confusion. When in doubt, it is useful to assess the clinical presentation and obtain an accurate medical history.

## 1. Introduction

Orbital surgery results in changes to the usual eye anatomy, which is often clearly apparent during radiology exams. As most physicians are not familiar with postoperative changes to the orbit, a proper radiological assessment of the globe and orbital abnormalities, often found incidentally when the eyes are included in the field during imaging of the head, may sometimes be challenging to radiologists and clinicians. As computed tomography (CT) is a valuable tool for assessing head trauma, familiarity with CT imaging features of incidental degenerative changes, globe implants, fillers, and traumatic globe irregularities is necessary for appropriate ophthalmological referral and correct diagnosis, as well as prevention of unnecessary workup.

Herein, we present a patient with head trauma who had surgery for retinal detachment with implantation of silicone encircling bands. Visibility of intraocular silicone oil and devices implanted for retinal detachment treatment could cause diagnostic confusion and be misleading for clinicians without the patient's medical history. The finding of silicone oil on CT may be recognized in the ophthalmology field; however, head/face CT imaging is used in many specialties, frequently exposing other clinicians to these and other findings in the eye. This case report may help clinicians recognize the imaging characteristics after ophthalmic surgery to prevent misdiagnosis and unnecessary workup.

## 2. Case Presentation

**Figure 1:**
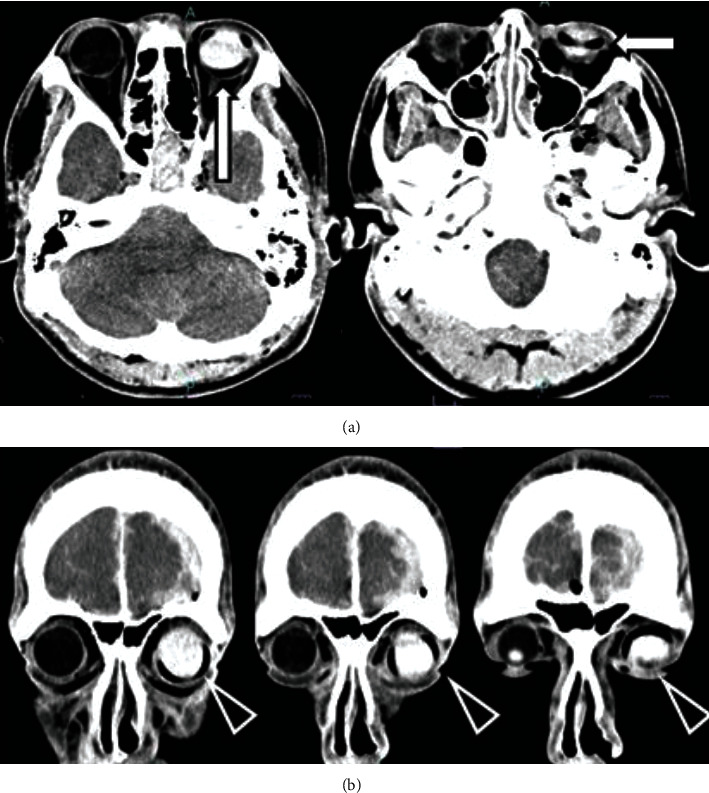
(a) Axial transverse CT and (b) coronal CT through the orbit. A low-density silicone rubber circling band (white arrow) and a well-defined, homogenous, hyperdense mass (black arrowhead) seen in the cross section were observed in the left eye.

An 18-year-old man came to the emergency department after losing consciousness following a high-speed traffic accident. His vital signs on arrival were as follows: respiratory rate of 20 breaths per minute, oxygen saturation of 99% with a nonrebreather mask, blood pressure of 210/130 mmHg, pulse of 107 beats per minute, and Glasgow Coma Scale score of six (E1V1M4). His body's physical examination showed a head bruise over the occipital area, a white area in his left eye, and anisocoria of the right pupil, with a diameter of 7 mm versus 2.5 mm for the left pupil. Following the initial assessment, brain CT scan showed a hyperdense, homogenous, well-defined mass in the left eye's posterior portion. A low-density band-like structure was found encircling the globe at the left eye's equator (Figure 1). A fracture of the left orbit wall with opacification of the adjacent ethmoid air, pneumocephalus, left acute subdural hematoma (ASDH), and subarachnoid hemorrhage were also present. He was admitted to the ICU after emergency craniotomy was performed for ASDH. As for the eyes, in-depth ophthalmological history revealed the patient had received previous vitreoretinal treatments for retinal detachment, including vitrectomy, silicone oil intraocular tamponade, and circumferential placement of a tightened silicone encircling band between the rectus muscles and the sclera when he was 16 years old. An urgent ophthalmologic assessment was initiated, and fundoscopy confirmed that the high-density area was silicone oil in the left eye. His eye examination findings were as follows: both corneas were clear, both anterior chambers were clear, and both lenses were clear. The intraocular silicone oil was not emulsified. The diagnosis for his right mydriatic eye was suspected to be oculomotor paralysis, and no further action was required regarding the left orbital region. On day 7, his right eye diameter improved to 2.5 mm. On day 18, magnetic resonance imaging (MRI) was performed for brain assessment because of continuous coma, and he was diagnosed with diffuse axonal injury. On day 28, he was transported for rehabilitation.

## 3. Discussion

About 2% to 6% of trauma patients are admitted to the hospital with ocular injuries [1]. Orbital traumas from indirect or direct injury to the optic nerve should be regarded as a surgical emergency. In these cases, early extraction of the foreign body is indicated, so vigilant clinical suspicion, careful physical examination, and selection of suitable imaging options are essential to avoid a misdiagnosis. Since trauma ranks second among causes of poor visual acuity and blindness, intervention should be considered.

Most imaging of the eye globe is completed with accompanying CT of the brain for numerous reasons, including trauma. Recent CT technology innovations that allow detailed globe visualization provide frequent, incidental findings of irregularities. Surgical materials are frequently used in ophthalmology, predominantly retinopexy materials for retinal detachment or artificial lenses for scleral buckling and cataracts. These materials are associated with different postoperative alterations that can be difficult to evaluate using MRI or CT [2]. As shown in our patient, intraocular silicone oil appears hyperdense relative to the extraocular muscles but hypodense relative to the orbital bone during CT for an initial assessment. Differential diagnosis of a well-defined, homogenous, hyperdense mass in the left eye may represent several possibilities, including vitreous hemorrhage, retinoblastoma, lymphoma, subchoroidal abscess, and metastatic tumor. Emergency physicians should become familiar with the unique imaging characteristics of intraocular silicone, as its high attenuation on CT may be mistaken for intraocular hemorrhage [3]. Since silicone oil has high attenuation on CT imaging, direct measurement of attenuation will reveal significantly higher attenuation values for silicone oil than for blood if eye surgical history is unavailable [4]. The Hounsfield unit (HU) measurement of silicone has been reported as high as 110 to 130 HU, whereas hemorrhage has an attenuation value of 50-90 HU [5].  Table 1 shows differential diagnoses of radiological presentation and density in HU on CT imaging (Table 1).

Band densities can differ depending on the types of bands used. The band can be identified crossing over and under the globe. Since silicone oil's prolonged presence in the eye or emulsification may affect the retina, optic nerve, and even the extraocular structures and result in complications like glaucoma, cataracts, and late corneal decompensation, prompt extraction of silicone oil is suggested immediately after it has fulfilled its purpose in tamponade [6]. However, a significant percentage of eyes that undergo silicone oil administration require long-term tamponade [7].

Silicone oil for ophthalmic applications almost exclusively comprises long-chain molecules showing a high-density area; conversely, breast implant silicone is a combination of both long and short chains appearing as an isodense area [4]. The oil is about as dense as the crystalline lens. Since silicone oil is not as dense as water, it can seem to move within the eye and float to the top, depending on the patient's head position. Low-density silicone bands encircle and indent the globe [8].

## 4. Conclusion

Intraocular liquid silicone infusion has been successfully used for vitreous replacement/internal tamponade and retinal reattachment. The radiological properties of silicone oil in the eye include high attenuation on CT, imitating a hemorrhage. This appearance is highly characteristic, and its origin can be confirmed by careful assessment of clinical presentation, obtaining an accurate medical history, and an ophthalmologist's review of CT images using a multidisciplinary approach.

## Figures and Tables

**Table 1:** Differential diagnoses on CT imaging.

	Silicone oil	Hydrogel	Hemorrhage	Normal eye
Radiological presentation	High attenuation	Moderately high attenuation	Moderate attenuation	Low attenuation
Density in HU	110 to 130 HU	110 HU	50 to 90 HU	20 HU

HU; Hounsfield unit.
